# Safety and Effectiveness of a 4-Week Diet on Low-Carb Ready-to-Eat Ketogenic Products as Preoperative Care Treatment in Patients Scheduled for Metabolic and Bariatric Surgery

**DOI:** 10.3390/nu16223875

**Published:** 2024-11-13

**Authors:** Biagio Santella, Monica Mingo, Alexander Papp, Mark Rice, Sonja Chiappetta, Pietro Calabrese, Fabrizia Calenda, Vincenzo Pilone, Luigi Schiavo

**Affiliations:** 1 Department of Medicine, Surgery and Dentistry “Scuola Medica Salernitana”, University of Salerno, 84081 Baronissi, Italy; bsantella@unisa.it (B.S.); mmingo@unisa.it (M.M.); 2 NBFC, National Biodiversity Future Center, 90133 Palermo, Italy; 3 P-Health Medical Solution, F.-W.-Raiffeisenstraße 1b, Elsbethen, 5061 Salzburg, Austria; med@drpapp.at; 4 Bariatric and Metabolic Surgery Unit, Department for General and Laparoscopic Surgery, Ospedale Evangelico Betania, 80147 Naples, Italy; nutrizioneonline@yahoo.it (M.R.); drschiappetta@gmail.com (S.C.); 5 Public Health Department, University of Naples Federico II, 80131 Naples, Italy; pietro.calabrese@unina.it (P.C.); fabrizia.calenda@unina.it (F.C.); vincenzo.pilone@unina.it (V.P.)

**Keywords:** metabolic and bariatric surgery, obesity, low-carb ketogenic diet, weight loss, left lateral liver section, preoperative care

## Abstract

Before metabolic and bariatric surgery (MBS), moderate weight loss and liver left lateral section (LLLS) volume reduction are desirable. Low-carb ketogenic diet-induced weight loss before MBS has been shown to have beneficial effects on the reduction in body weight (BW) and LLLS. However, the nutritional protocol of low-carb ketogenic diet may be hard to keep for prolonged periods due to the lack of sweet taste. Furthermore, transitioning to a low-carb ketogenic diet can cause people to crave foods that are restricted in the ketogenic diet, such as cookies, bread, pasta, and bagels. Therefore, many ready-to-eat low-carb ketogenic products (RLCKP) that mimic carbohydrate-rich foods despite a low-carb composition have been provided to make it easier for the patients to adopt a low-carb lifestyle. To date, there are no studies describing the dietary protocol for efficient and safe use of pre-operative RLCKP in terms of weight and LLLS volume reduction in patients with obesity scheduled for MBS. Therefore, the aim of this study was to assess the safety and effectiveness of a 4-week diet using RLCKP in reducing BW and LLLS volume in patients with obesity scheduled for MBS. Patients with obesity (*n* = 42) with a mean body mass index (BMI) of 42.4 ± 9.2 kg/m^2^ scheduled for MBS underwent a 4-week preoperative RLCKP diet intervention. Their weight, LLLS volume, and biochemical and metabolic parameters were measured before and after the diet. Patient compliance was assessed by the presence of ketonuria and weight loss. Qualitative methods (5-point Likert questionnaire) were used to measure diet acceptability and side effects. All patients completed the study. We observed highly significant decreases in BW (−6.5%, *p* < 0.001), and LLLS volume (−22.3%, *p* < 0.001) and an amelioration of patient clinical status. All patients showed a high frequency of acceptability and compliance in following the diet. No adverse side effects were reported. Based on our findings, we were able to support the hypothesis that a 4-week preoperative RLCKP diet is safe and effective in reducing BW, and LLLS volume in patients with obesity scheduled for MBS.

## 1. Introduction

Metabolic and bariatric surgery (MBS) is widely acknowledged as the most effective and enduring solution for addressing morbid obesity [1]. Prior to undergoing MBS, it is beneficial for patients to achieve moderate weight loss (WL) along with a reduction in the volume of the liver’s left lateral section (LLLS) [2]. Individuals suffering from morbid obesity frequently present with an enlarged LLLS which can complicate surgical procedures. Such complications may result in prolonged operative times, increased risks of intraoperative bleeding, higher rates of conversion to open surgery, issues related to anastomosis, and suboptimal bariatric anatomy that may negatively impact long-term outcomes [2,3,4]. Consequently, preoperative interventions aimed at decreasing LLLS volume prior to laparoscopic MBS could yield advantages for both patients and surgical teams [5].

To promote moderate WL and reduce LLLS volume before MBS, various dietary strategies have been established over time [6]. Among these strategies, low-carb ketogenic diets (LCKDs) are often recommended [6,7]. Research conducted by Schiavo et al. has shown that a 4-week preoperative LCKD is safe and effective in achieving a body weight reduction of −9.2% alongside a -19.8% decrease in LLLS volume among patients preparing for MBS [8]. Additionally, Pilone et al. assessed the safety, effectiveness, and acceptability of a specialized Keto-Station kit protocol for LCKD among pre-MBS patients, confirming its efficacy in reducing total body weight and LLLS volume [9]. Furthermore, Albanese et al. compared surgical outcomes and weight loss between two groups following different preoperative diets—low-calorie versus ketogenic—and found that those adhering to the ketogenic diet experienced superior surgical outcomes including reduced drainage output, improved postoperative hemoglobin levels, and shorter hospital stays [10]. However, these beneficial effects can be diminished by inadequate adherence to dietary guidelines [11]. Maintaining a prolonged commitment to an LCKD can prove challenging due to restricted sweet flavor options; transitioning to such a diet often results in cravings for limited foods like cookies, bread, pasta, and bagels [12]. To mitigate this issue, numerous ready-to-eat low-carb ketogenic products (RLCKP) have been created to replicate the texture and flavor of conventional carbohydrate-rich foods while remaining low in carbohydrates. These products facilitate easier adherence to an LCKD for patients [13] with evidence indicating that RLCKP usage improves compliance with ketogenic dietary regimens [14,15,16]. Currently, no research exists investigating the efficacy or safety of preoperative RLCKP dietary regimens concerning weight loss or reductions in LLLS volume among individuals with obesity scheduled for MBS. Therefore, this study aims to assess the safety and effectiveness of a 4-week regimen incorporating RLCKP designed to reduce body weight and LLLS volume in patients preparing for MBS.

## 2. Patients and Methods

### 2.1. Study Design and Patients Selection

A multicenter prospective cohort study was undertaken involving patients with obesity who were scheduled for metabolic and bariatric surgery (MBS) from January to November 2023 across three European obesity centers located in Salerno, Napoli, and Salzburg. The participant group consisted of 42 individuals with obesity, comprising 15 males and 27 females, with an average age of 42 years (±11.6). The mean total body weight was recorded at 118.4 kg (±29.3), and the average body mass index (BMI) was calculated as 42.4 kg/m^2^ (±9.2). All participants met the criteria established by the International Federation for Surgery of Obesity regarding surgical intervention for morbid obesity. Inclusion criteria specified a BMI of ≥40 kg/m^2^ or ≥35 kg/m^2^ accompanied by at least one comorbidity (such as diabetes, hypertension, sleep apnea or heart disease), an age range between 18 and 65 years, and a health status conducive to adhering to a ketogenic diet—specifically normal renal function without pregnancy or lactation. Exclusion criteria included:

1. Significant challenges in altering dietary habits;
2. Inability to comply with the RLCKP diet due to religious beliefs or chewing/swallowing difficulties;
3. Presence of food allergies or intolerances, as well as digestive or inflammatory bowel diseases such as Crohn’s disease or ulcerative colitis;
4. A serum creatinine level exceeding 1.8 mg/dL or liver enzyme levels (glutamic oxaloacetic transaminase [GOT] or glutamic pyruvic transaminase [GPT]) surpassing three times the upper limit of normal.

The study protocol received approval from the University Ethical Committee (EK Nr: 1023/2024, Approval Date: 25 July 2024). All procedures adhered to both institutional and national ethical standards pertaining to research, consistent with the principles outlined in the Declaration of Helsinki and its subsequent amendments. Written informed consent was secured from all participants after they were briefed on the study’s aims and methodologies. Participants were provided complimentary RLCKP products (My Life-Changer Foodfast^®^, Schwaz, Austria) by KETTOX GmbH (Wien, Austria). The RLCKP protocol was developed by P-Health GmbH (Elsbethen, Austria), which tailored programs according to each individual’s clinical profile. During their initial visit, participants received a comprehensive menu that detailed allowed and prohibited foods alongside components necessary for following the RLCKP regimen.

### 2.2. Dietary Intervention

**Figure 1:**
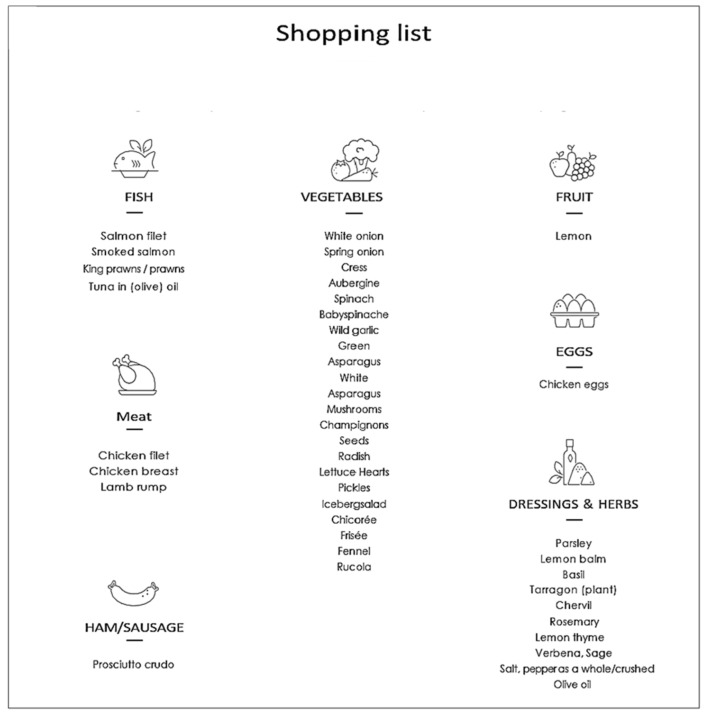
Shopping lists.

Before starting the nutritional treatment, candidates received comprehensive nutritional counseling and resources aimed at enhancing their understanding and compliance with the RLCKP protocol. These resources included shopping lists that detailed all permissible foods along with recommended daily meal plans and recipes. The lists promoted the consumption of unprocessed foods which included meats, poultry, and fish varieties such as salmon and tuna, in addition to cooked green vegetables and cold cuts like dried beef and eggs. Participants were also allowed to incorporate up to three tablespoons of olive oil per day, a maximum of 14 g of butter, and an intake of 10 g of nuts. A thorough list of acceptable foods can be found in Figure 1.

**Figure 2:**
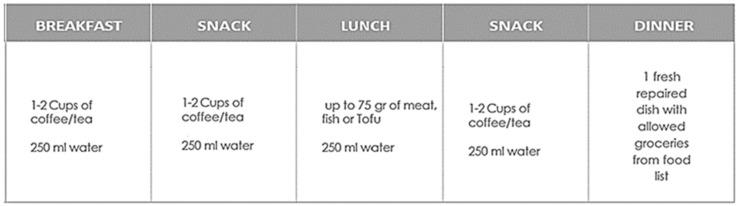
Daily Food Intake.

As provided by the menu plan (Figure 2), 1–2 cups of coffee or tea are intended for breakfast and afternoon snack time.

The drinking behavior for the first phase of this study is scheduled in a way that patients are requested to drink the specified drinks of the weekly menu plan of RLCKP (Figure 1 and Figure 2) and additionally about 1.5 L of water a day plus 40 mL of the Cellviva liquids© formula Mydrain & 40 mL Mymetabol) which comprehend phytoextracts. The ingredients are illustrated in Table 1 and Table 2.

The RLCKP method calls for five meals per day in total, four of which consist of nutraceutical portions from the supplier Kettox GmbH with at least 14 g of protein and a 5th meal that must be prepared independently, preferably using food that is of high quality, fresh, and should not be pre-cooked. This is intended to help the individual patient manage their intake of various foods and macronutrients and to learn sustainably which foods can continue to be consumed without problems after the diet period.

The ingredients of nutraceuticals are plant and animal proteins, collagen peptides, resistant starch, prebiotic fiber, Acacia fibers, inulin fiber, and gluten-free. PP (Protein Portion) or nutraceutical portion is a measure of a certain dish (1 protein bar, 1 shake, 1 soup or 35 grams of special protein pasta, cookies, etc., all from Kettox, with high protein and very low carbohydrates) that contains at least 15 g of proteins.

**Figure 3:**
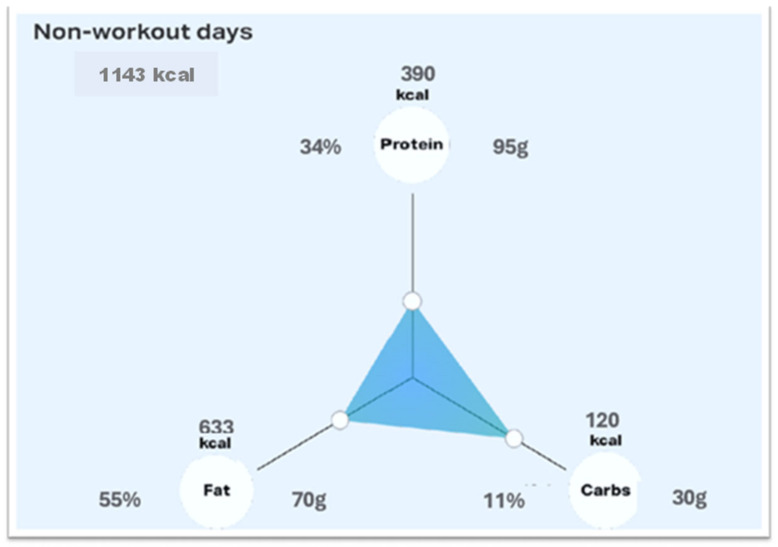
The macronutrients are calculated individually for every patient.

The menu plans of each phase are designed for patients to always have a sufficient food intake to cover their daily protein needs. In assistance, patients were provided with a brochure containing a weekly “shopping list” (Figure 1) as well as a menu plan to provide detailed information and constitute a suitable template for all patients during the dietary intervention. The RLCKP protocol was set to endure 4 weeks and intends to completely eradicate the intake of sugars and carbohydrates in the metabolic system. The aim thereof is the achievement of a ketosis metabolic state at which the human metabolism switches its energy supply from intake of sugars and carbohydrates to self-metabolization of fatty acids in the liver to provide a source of energy in the form of ketone bodies. The macronutrients are calculated individually for every patient and visualized (Figure 3). The eating period is restricted to a maximum of 12 h per day. Each participant was followed for four weeks, and a daily notice was taken of the compliance/adherence to the diet.

### 2.3. Assessment of Dietary Compliance, Acceptability, and Side Effects

Patient adherence was evaluated through the use of ketonemia and validated by monitoring weight reduction. Each patient provided a urine sample at baseline as well as during each weekly outpatient appointment. Qualitative approaches were employed to assess dietary side effects and overall acceptability. During the weekly counseling sessions, patients were requested to fill out a questionnaire that gauged diet acceptability and reported symptoms such as hunger, nausea, vomiting, headaches, halitosis, and constipation on a 5-point Likert scale, referencing the framework established by Colles et al. [5,17]. A qualified nutritionist meticulously reviewed all submitted questionnaires to ensure they were accurate and complete.

### 2.4. Anthropometric, Laboratory Determinations, and LLLS Volume Measurements

In all subjects, body weight (kg) and height (cm) were measured under standardized conditions which included a fasting state and participants wearing light street clothing without heavy items such as shoes. Height was assessed using a mechanical measuring tape while body weight (BW) was evaluated with a digital scale that has a maximum capacity of 250 kg. Measurements for BW were taken at the baseline and subsequently on a weekly basis throughout the four-week follow-up period.

The blood tests conducted included analyses of liver enzymes (GOT, GPT, and gamma-glutamyl transferase [GGT]), glycemic profile indicators (glucose and insulin levels), renal function metrics (creatinine and glomerular filtration rate [GFR]), ketone bodies in the blood, iron levels, hemoglobin A1C, lipid profile components (total cholesterol, high-density lipoprotein [HDL], and triglycerides), homeostasis model assessment (HOMA) index, along with concentrations of ions such as sodium (Na^+^), chloride (Cl^−^), and potassium (K^+^).

All laboratory analyses were carried out in an accredited facility that adhered to both internal and external quality control protocols. The testing utilized reagents supplied by the manufacturer while strictly following their guidelines. Results were then compared against established clinical cutoff values. Blood samples were obtained at baseline and retested after four weeks.

To measure the volume of the LLLS, an ultrasound system was employed. Consistent with methodologies from previous studies [18], LLLS volume was calculated using measurements of the transverse axis, super inferior axis, and midsection of the anteroposterior axis (“thickness”), under the assumption that its shape approximated that of a half-rectangular parallelepiped.

### 2.5. Statistical Analysis

Data are presented as the mean ± standard deviation (SD) for continuous variables and as percentages for dichotomous variables. The normality of the data was evaluated using the Shapiro–Wilk test, revealing that most variables exhibited a non-normal distribution. Within-group comparisons were conducted using the Wilcoxon signed-rank test on paired samples. A significant level of 5% was established for all analyses, with differences deemed statistically significant at *p* < 0.05. The statistical analysis was executed utilizing XLSTAT (Lumivero, 2024), a statistical and data analysis solution based in Paris, France. Additionally, *p*-values less than 0.001 were simply reported as *p* < 0.001 to denote their high statistical significance.

## 3. Results

### 3.1. Changes in Total Body Weight, BMI, and LLLS Volume

A total of 42 patients (15 males and 27 females) were enrolled in this study. The mean age was 42 (±11.6) years. The total body weight, BMI, and the LLLS volume at baseline and after a 4-week course of preoperative RLCKP diet are shown in Table 3.

### 3.2. Biochemical Parameters Results

As reported in Table 4, concerning the clinical characteristics of the study patients at baseline and after a 4-week course of preoperative RLCKP diet, we found a significant amelioration of the general clinical status.

### 3.3. Compliance, Acceptability, and Side Effects of RLCKP Diet

**Figure 4:**
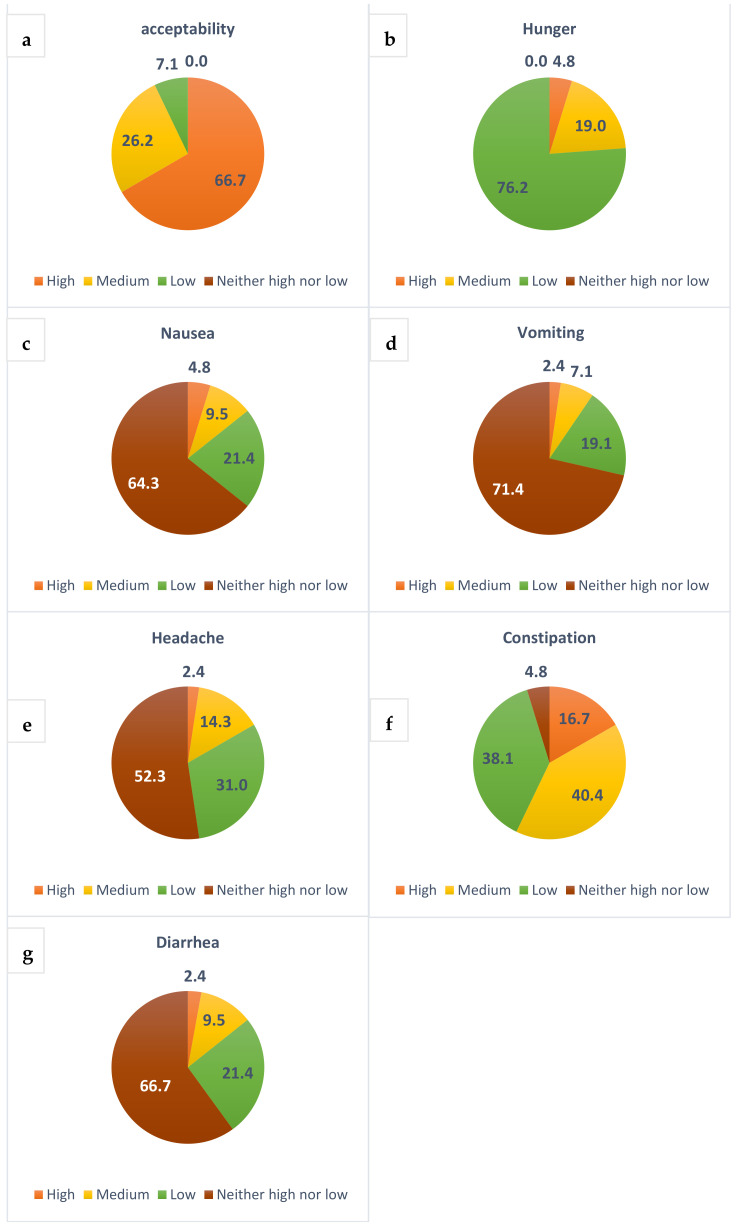
Self-reported rates regarding the acceptability of the diet (**a**) and the occurrences of hunger (**b**) nausea (**c**) vomiting (**d**) headaches (**e**) constipation (**f**) and diarrhea (**g**) over the four-week period of the pre-operative RLCKP diet.

We conducted an evaluation of the median values related to dietary acceptability and the occurrence of hunger, nausea, headaches, and bowel function by utilizing self-reported questionnaires administered from week 1 to week 4 of the diet. The majority of the participants demonstrated a high level of acceptability (see Figure 4a). Hunger was reported frequently by 4.8% of the patients (refer to Figure 4b), while instances of nausea (Figure 4c) and vomiting (Figure 4d) were infrequently noted during the diet period. Headaches, likely attributed to the presence of ketones, were reported frequently by 16% of participants (see Figure 4e). Lastly, medium constipation was the most reported gastrointestinal issues (refer to Figure 4f,g).

## 4. Discussion

Based on our findings, we were able to support the hypothesis that a 4-week preoperative RLCKP diet is safe and effective in reducing BW and LLLS volume in patients with obesity scheduled for MBS.

Among the various MBS techniques, sleeve gastrectomy and Roux-en-Y gastric bypass are the most performed [19], and they are usually performed laparoscopically [20]. However, laparoscopic MBS in patients with obesity can be challenging as the enlarged LLLS volume can obstruct the surgical field and increase the risk of complications, such as anastomotic leakage, bleeding, and prolonged conversion rate and surgical time [2,3,4].

As a consequence, as reported by the Italian Society of Obesity Surgery and Metabolic Diseases guidelines [1], the American Society for Metabolic and Bariatric Surgery position statement on preoperative patient optimization before MBS [21], and the Enhanced Recovery After Surgery (ERAS^®^) Society guideline for perioperative care in bariatric surgery [22], the pre-operative reduction in BW and liver volume are recommended in patients who are candidates for MBS, especially in the presence of BMI > 40 kg/m^2^.

Low-carb ketogenic diet-induced weight loss before MBS has been shown to have beneficial effects on the reduction in body weight (BW) and LLLS [23,24]. Herein, we observed significant decreases in BW and LLLS volume that were comparable to those observed in previous studies [8,9,10,23,24].

From the standpoint of the bariatric surgeon, the decrease in body weight and LLLS volume in patients with obesity scheduled for MBS is particularly noteworthy. The two primary factors that determine the technical challenges that may arise during any bariatric treatment are the size of the liver’s segments II and III as well as the kind and quantity of intraabdominal fat distribution. Since the majority of the fat is concentrated around the gastrosplenic ligament, the larger omentum, and the esophagogastric junction, these patients are in fact quite challenging to operate on. The approach to the stomach may be very challenging under these circumstances. Additionally, under these circumstances, the liver may take up the majority of the left upper quadrant’s surgical field and is prone to bleeding when moving to expose the stomach [3,4,5].

In this study, a 4-week preoperative RLCKP diet reduced the baseline LLLS volume by a mean of 22.3% which was similar to or more pronounced than in the literature [5,17,18,25,26,27,28,29,30].

We also found that patients were compliant with the diet protocol based on consistent weight loss and presence of ketonuria in accordance with other studies that attended weight loss using a very low-carb ketogenic diet [24,31,32,33].

Regarding the clinical status of patients, the RLCKP diet resulted in a significant improvement in the glycemic and lipid profile. These data confirm what was reported in a recent meta-analysis conducted by Alarim et al. who showed that the low-carb ketogenic diet is superior to other nutritional strategies in terms of improvements in lipid profile and glycemic control [34], even in patients living with obesity and type 2 diabetes [35,36].

This effect is explained, at least in part, by the biochemistry of ketogenesis which, as is well known, represents a biochemical strategy that the human metabolism uses in conditions of dietary carbohydrates deficiency to produce the glucose necessary for the correct cellular physiology from the fats of the white adipose tissue.

Nonetheless, there is ongoing discussion regarding the long-term appropriateness, safety, effectiveness, and possible advantages of a low-carbohydrate ketogenic diet in comparison to traditional dietary strategies for managing diabetes. The implementation of recommendations pertaining to a ketogenic diet within clinical practice frequently encounters obstacles due to the absence of a clear definition. This ambiguity limits its potential as an optimal therapeutic choice for diabetes management [35].

Furthermore, a considerable proportion of patients were satisfied with the RLCKP diet according to evaluations of tolerability and acceptability through self-answered questionnaires. All tested patients had a high frequency of acceptability and low rates of hunger and secondary effects.

This study has some limitations, including the small number of patients studied and the short-term follow-up that did not allow definitive conclusions to be drawn. Furthermore, we are aware that other methods, such as computed tomography (CT) or magnetic resonance, would certainly have given us more accurate measurements of the LLLS volume. However, patients with obesity often have difficulty accessing such techniques due to the physical limitations of the equipment (e.g., the strength of the table and the size of the hole). In addition, CT is not cost-effective, and radiation exposure associated with CT scans can be problematic; therefore, it is often not suitable for screening large groups of individuals. However, the results for LLLS volume measurements by ultrasound appeared to be reliable and reproducible.

The main strengths of the present study are:

- The RLCKP diet could represent a possible alternative to other low-carb ketogenic diets, in particular in low adherent patients. Indeed, the nutritional protocol of the classic low-carb ketogenic diet may be hard to keep for prolonged periods due to the lack of sweet taste. Furthermore, transitioning to a low-carb ketogenic diet can cause people to crave foods that are restricted to the ketogenic diet, such as cookies, bread, pasta, and bagels. Therefore, the use of a RLCKP that mimics carbohydrates foods despite a low-carb composition could make it easier for patients to adopt a low-carb eating lifestyle.
- To the best of our knowledge this is the first study describing the dietary protocol for efficient and safe use of pre-operative RLCKP in terms of weight and LLLS volume reduction in patients with obesity scheduled for MBS.
- The RLCKP protocol could be useful in patients showing a poor adherence in following the standard ketogenic diet in order to increase the patient’s motivation and to avoid and/or minimize delays of surgical treatment.
- The RLCKP diet is an effective and safe treatment before MBS in reducing BW and LLLS volume and in improving patient’s clinical status.
- Regarding safety, no important side effects were reported.

## 5. Conclusions

Based on our findings, we were able to support the hypothesis that a 4-week preoperative RLCKP diet is safe and effective in reducing BW and LLLS volume in patients with obesity scheduled for MBS. Further studies are needed to confirm these preliminary data.

## Figures and Tables

**Table 1:** Cellviva Liquids©—Mymetabol.

MYMETABOL		
Nutritionally Active Substances in Plant Extracts	Per Daily Dose (60 mL)	Per 100 mL
Rhubarb	55 mg	92 mg
Spearmint	47 mg	78 mg
Dandelion root	47 mg	78 mg
Radish	40 mg	67 mg
Burdock root	29 mg	48 mg
Field horsetail	29 mg	48 mg
Licorice	29 mg	48 mg
Artichoke	29 mg	48 mg
Brown seaweed powder	25 mg	42 mg
Crushed anise seed	22 mg	36 mg
Elderflower	22 mg	36 mg
Melissa leaf	22 mg	36 mg
Gentian root	19 mg	31 mg
Crushed juniper barriers	19 mg	31 mg
Parsley	19 mg	31 mg
Ginseng root	9 mg	16 mg
Cinchona bark	9 mg	16 mg

**Table 2:** Cellviva Liquids©—Mydrain.

MYDRAIN		
Nutritionally Active Substances in Plant Extracts	Per Daily Dose (60 mL)	Per 100 mL
Horsetail herb	52 mg	87 mg
Asparagus root	52 mg	87 mg
Birch leaves	41 mg	69 mg
Marshmallow leaves	38 mg	64 mg
Couch grass root	36 mg	60 mg
Corn silk	36 mg	60 mg
Elderberry	33 mg	55 mg
Karkade hibiscus	33 mg	55 mg
Aniseed crushed	32 mg	53 mg
Dandelion root	31 mg	51 mg
Bearberry leaves	31 mg	51 mg
Fennel	22 mg	36 mg

**Table 3:** Total body weight, BMI, and LLLS volume at baseline and after a 4-week course of preoperative RLCKP diet.

Clinical Characteristics	Baseline Mean ± SD	Follow-Up Mean ± SD	Δ%	*p*
Patients (*n*)	42	42		
Total body weight (kg)	118.4 ± 29.3	110.7 ± 27.5	−6.5	<0.001
BMI (kg/m^2^)	42.2 ± 9.2	38.6 ± 9.3	−8.6	<0.001
Lateral left liver section (cm^3^)	635.1 ± 30.3	493.4 ± 41.3	−22.3	<0.001

BMI body mass index.

**Table 4:** Clinical characteristics of the study patients at baseline and after a 4-week course of preoperative RLCKP diet. HDL = high density lipoprotein; LDL = low density lipoprotein; GOT = glutamic oxaloacetic transaminase; GPT = glutamic pyruvic transaminase; GGT = gamma-glutamyl transferase; GFR = glomerular filtration rate.

Clinical Characteristics	Baseline Mean ± SD	Follow-Up Mean ± SD	Clinical Cut-Off Values	Δ%	*p*
Patients (*n*)	42	42			
Glucose (mg/dL)	93.1 ± 14.7	86.9 ± 12.3	74–106	−6.6	<0.001
Insulin (mU/L)	16.9 ± 11.4	13.9 ± 11.8	1.9–16	−17.7	<0.001
HOMA Index	4.1 ± 3.3	3.1 ± 2.99	0.23–2.5	−24.4	<0.001
Hemoglobin A1C (%)	5.6 ± 0.56	4.8 ± 1.20	4–6	−14.3	<0.001
Creatine (mg/dL)	0.8 ± 0.18	0.78 ± 0.14	0.72–1.18	−2.5	0.007
BUN (mg/dL)	22.6 ± 8.2	24.4 ± 7.83	10–24	+7.9	0.03
GFR (mL/min)	171.6 ± 99.8	167.2 ± 83.4	90–120	−2.6	0.34
Iron (µg/dL)	84.7 ± 28.4	86.3 ± 28.8	60–160	+1.9	0.62
Total cholesterol (mg/dL)	202.7 ± 37.8	185.7 ± 30.9	<200	−8.4	<0.001
HDL (mg/dL)	60.6 ± 26.7	54.5 ± 14.7	>35	−10	0.08
Triglycerides (mg/dL)	142.3 ± 59.3	120.2 ± 47.2	<150	−15.5	<0.001
GOT (U/L)	27.4 ± 13.6	24.2 ± 9.30	5–50	−11.7	0.18
GPT (U/L)	31.9 ± 18.9	30.9 ± 21.4	5–50	−3.1	0.61
GGT (U/L)	23.2 ± 9.75	19.5 ± 9.7	0–55	−15.76	<0.001
Ketonemia (mmol/L)	0.87 ± 0.08	1.2 ± 0.96	<0.6	+37.8	<0.001
Na^+^ (mEq/L)	141.1 ± 2.79	140.5 ± 3.56	135–146	−0.4	0.13
K^+^ (mEq/L)	4.4 ± 0.44	4.1 ± 0.42	3.5–5.1	−6.8	0.91
Cl^−^ (mEq/L)	103.1 ± 3.4	100 ± 16.0	101–110	−3.0	0.21

## Data Availability

The data included in this manuscript derived from the University database. We are not authorized to share the data with third party organizations. However, the corresponding author is available to provide any explanation to the Editor if requested.
